# The symphony of maize signaling quartet defending against gray leaf spot

**DOI:** 10.1007/s44154-024-00157-x

**Published:** 2024-03-14

**Authors:** Ping Wang, Ping He

**Affiliations:** 1 Department of Science, China Agricultural University, Beijing, 100193 China; 2 Department of Molecular, Cellular, and Developmental Biology, University of Michigan, Ann Arbor, MI 48109 USA

**Keywords:** Receptor-like kinases, Receptor-like cytoplasmic kinases, NADPH oxidase, Grey leaf spot, Pattern-triggered immunity

## Abstract

In plant immunity, a well-orchestrated cascade is initiated by the dimerization of receptor-like kinases (RLKs), followed by the phosphorylation of receptor-like cytoplasmic kinases (RLCKs) and subsequent activation of NADPH oxidases for ROS generation. Recent findings by Zhong et al. illustrated that a maize signaling module comprising ZmWAKL-ZmWIK-ZmBLK1-ZmRBOH4 governs quantitative disease resistance to grey leaf spot, a pervasive fungal disease in maize worldwide, unveiling the conservation of this signaling quartet in plant immunity.

## Main text

Plant receptor-like kinases (RLKs) are important cell surface pattern recognition receptors (PRRs), recognizing a plethora of microbe- and danger-associated molecular patterns (MAMPs/DAMPs), ultimately orchestrating pattern-triggered immunity (PTI). Plant genomes contain hundreds of RLKs with diverse ectodomain (ECD) architectures, such as leucine-rich repeat (LRR), lysine motif (LysM), lectin motif, and malectin-like domain (Hou et al. 2021). Wall-associated kinases (WAKs) and WAK-like kinases (WAKLs) are a unique group of RLKs with extracellular galacturonan-binding (GUB) domains, which likely link to the cell wall (Stephens et al. 2022). WAKs and WAKLs emerge as formidable guardians against pathogens with diverse lifestyles (Stephens et al. 2022). For example, cotton GhWAK7A mediates resistance to Verticillium and Fusarium wilts by modulating the fungal chitin sensory complex (Wang et al. 2020).

A recent study in Nature Genetics by Zhong et al. illustrated a saga of maize WAKL-mediated defense against gray leaf spot (GLS), caused by *Cercospora zeae-maydis* and *Cercospora zeina*, a major fungal disease in maize worldwide (Zhong et al. 2024). Through the meticulous fine mapping of the major quantitative disease resistance (QDR) locus and comprehensive transgenic assays, ZmWAKL was found to be the major gene for conferring resistance against GLS, with ZmWAKL^Y^ as the resistant isoform and ZmWAKL^Q^ as the susceptible isoform. While the intracellular domain (ICD) of ZmWAKL^Y^ and ZmWAKL^Q^ remains highly conserved, their ECDs, particularly the GUB domain, diverge significantly. ECDs in RLKs play a crucial role in ligand binding. The data suggest that ZmWAKL^Y^ and ZmWAKL^Q^ likely have differences in perceiving MAMPs from pathogens or DAMPs from plants. This hypothesis was corroborated by an elegant chimeric gene experiment in which the fuse of ECD from ZmWAKL^Y^ and ICD from ZmWAKL^Q^ conferred the GLS resistance (Zhong et al. 2024). Similarly, the variation between resistant and susceptible isoforms of Rlm9, an oilseed WAKL involved in race-specific resistance against *Leptosphaeri maculans*, is concentrated in the ECD GUB domain (Larkan et al. 2020). In addition, unlike ZmWAKL^Q^, ZmWAKL^Y^ was self-associated via the GUB domain, suggesting the formation of homodimers of ZmWAKL^Y^. The ICD of ZmWAKL^Y^, not ZmWAKL^Q^, exhibited kinase activity both in vitro and in vivo, substantiating the importance of ZmWAKL^Y^ in transducing immune signaling (Zhong et al. 2024). Notably, the phosphorylation of ZmWAKL^Y^ was enhanced after *C. zeina* infection, supporting the physiological importance of ZmWAKL^Y^ phosphorylation.

By performing an immunoprecipitation-mass spectrometry assay, Zhong et al. further demonstrated that ZmWIK, an LRR-RLK, interacted with ZmWAKL and was important in conferring GLS resistance. Both the ECD and ICD of ZmWIK preferentially interacted with ZmWAKL^Y^ compared to ZmWAKL^Q^ (Zhong et al. 2024). Similar to other RLK complexes, the transphosphorylation was observed between ZmWIK and ZmWAKL. Apparently, ZmWIK was more strongly phosphorylated than ZmWAKL when these two proteins were mixed, suggesting a potential role for ZmWIK in amplifying ZmWAKL phosphorylation in the signal relay process. With RLK receptor and coreceptor dimerization and transphosphorylation serving as the cornerstone of plant PTI (Hou et al. 2021), the authors proposed that ZmWIK is a coreceptor of ZmWAKL. Yet, intriguing questions persist, including the regulation of ZmWIK-ZmWAKL dimerization in response to pathogen invasion and the potential divergence in ZmWIK sequences and expression between GLS-resistant and susceptible maize varieties.

Receptor-like cytoplasmic kinases (RLCKs) are convergent signaling regulators downstream of multiple PRRs in relaying multiple intracellular signaling events, including ROS burst, calcium influx, and MAPK activation (Lin et al. 2013; Liang and Zhou 2018). Similarly, maize RLCK ZmBLK1 was associated with both ZmWIK and ZmWAKL. Apparently, the association between ZmBLK1 and ZmWIK was stronger than the ZmBLK1-ZmWAKL association, supporting the role of ZmWIK being the coreceptor (Zhong et al. 2024). Moreover, ZmBLK1, ZmWAKL, and ZmWIK form a complex at the plasma membrane, underscoring their collaborative efforts in immune signaling. Furthermore, ZmWIK strongly phosphorylated ZmBLK1, and this phosphorylation was further increased when ZmWAKL^Y^ was present (Zhong et al. 2024). This aligns with the interaction and phosphorylation of Arabidopsis RLCK BIK1 by the PRR coreceptor BAK1 (Lin et al. 2014).

A key function of RLCKs in plant immunity is to regulate ROS generation by phosphorylating NADPH oxidases, specifically the respiratory burst oxidase homologs (RBOHs) (Liang and Zhou 2018). Zhong et al. found that ZmRBOH4, one of the six RBOHs in maize, was highly expressed and induced by pathogens in leaves. The *ZmRBOH4* knocked-out lines exhibited compromised *C. zeina*-induced ROS levels and were susceptible to GLS, paralleling the vulnerability observed in *Zmwik* null mutants (Zhong et al. 2024). Biochemistry assays revealed that ZmBLK1 interacted and phosphorated the N-terminal region of ZmRBOH4. Moreover, the phosphorylation of ZmRBOH4 by ZmBLK1 was enhanced in the presence of ZmWAKL^Y^ and ZmWIK, supporting that ZmRBOH4 is a component of ZmWAKL^Y^-ZmWIK-ZmBLK1 complex orchestrating GLS resistance in maize (Zhong et al. 2024).

**Fig. 1:**
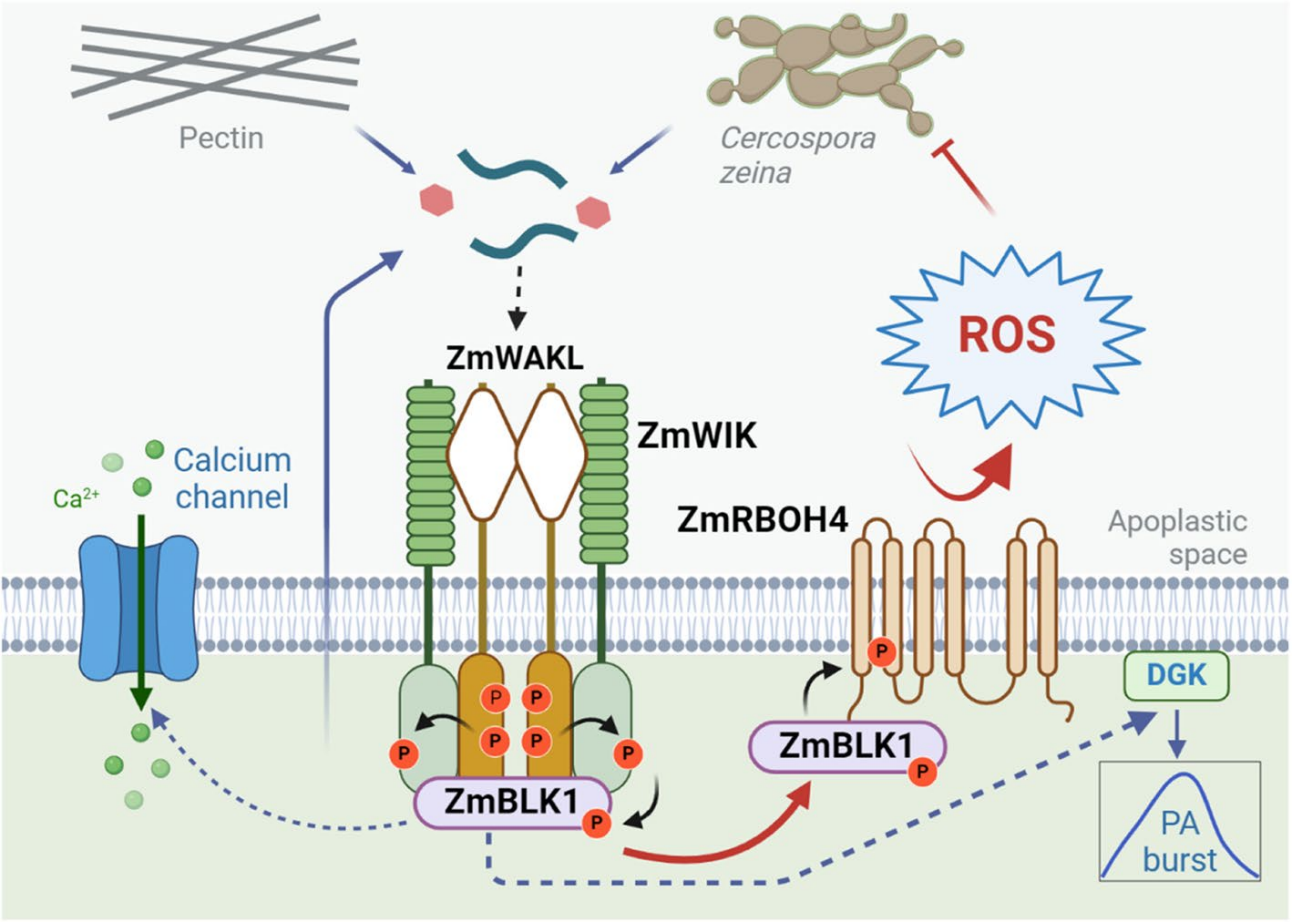
The proposed model of the ZmWAKL–ZmWIK–ZmBLK1–ZmRBOH4-mediated immunity. Upon *C.* z*eina* invasion, the ligand molecule from the pathogen, plant cell wall pectin, or secreted protein from cytosol is sensed by the ZmWAKL homodimer and induces ZmWAKL phosphorylation. ZmWIK functions as a coreceptor of ZmWAKL and is phosphorylated by ZmWAKL. ZmBLK1 is the RLCK that interacts with and is phosphorylated by both ZmWAKL and ZmWIK for signaling relay. The NADPH oxidase ZmRBOH4 is the substrate of ZmBLK1 and generates the ROS burst upon ZmBLK1 phosphorylation, culminating in GLS resistance. It is possible that ZmBLK1 also activates other substrates, such as calcium channels and diacylglycerol kinases (DGKs), for calcium influx and phosphatidic acid (PA) burst, conferring broad resistance against pathogens

Collectively, in this groundbreaking discovery, the authors uncover a conserved signaling quartet that serves as the linchpin in maize's defense against GLS. The quad, comprising ZmWAKL, ZmWIK, ZmBLK1, and ZmRBOH4, emerges as a masterful conductor orchestrating maize's immune defense mechanism (Fig. 1). By drawing parallels with analogous modules in other plant species, this study illuminates the evolutionary conservation of pivotal signaling pathways across diverse plant lineages. A pivotal aspect of this discovery lies in the recognition of ZmWAKL as a quantitative disease-resistance gene, a feat often hindered by the challenges associated with dissecting and verifying quantitative traits genetically. Nevertheless, by unraveling the intricate workings of the ZmWAKL–ZmWIK–ZmBLK1–ZmRBOH4 module, this study not only enhances our understanding of maize immunity but also unveils promising avenues for engineering crops with enhanced disease resistance.

WAKs/WAKLs play versatile roles during plant–microbe interactions, ranging from the detection of potential patterns from plants or microbes, modulation of PRR complexes, function of signaling components, and biosynthesis of cellulose and secondary metabolite (Stephens et al. 2022). As such, probing the precise role of ZmWAKL in perceiving either MAMPs from *C. zeina* or DAMPs released or induced in plants upon infection is an intriguing avenue for future research. Similarly, the possibility of ZmWIK serving as the *bona fide* PRR within the ZmWAKL–ZmWIK–ZmBLK1–ZmRBOH4 module cannot be discounted. Furthermore, in addition to their involvement in regulating ROS burst, Arabidopsis RLCKs have been shown to modulate cytosolic calcium influx and phosphatidic acid burst in plant immunity (Fig. 1) (Tian et al. 2019; Kong et al. 2024). Exploring whether the ZmWAKL–ZmWIK–ZmBLK1–ZmRBOH4 module extends its regulatory reach to other signaling events in plant immunity could provide valuable insights into its contribution to broad-spectrum resistance.

## Data Availability

Not applicable.

## Abbreviations

RLKs: Receptor-like kinases
PRRs: Pattern recognition receptors
MAMPs/DAMPs: Microbe-/danger-associated molecular patterns
PTI: Pattern-triggered immunity
ECD: Ectodomain
LRR: Leucine-rich repeat
LysM: Lysine motif
WAKs: Wall-associated kinases
WAKLs: WAK-like kinases
GUB: Galacturonan-binding
GLS: Gray leaf spot
QDR: Quantitative disease resistance
ICD: Intracellular domain
RLCKs: Receptor-like cytoplasmic kinases
RBOHs: Respiratory burst oxidase homologs
